# Timing and body condition of dichromatic Black Redstarts during autumn migration

**DOI:** 10.1002/ece3.2911

**Published:** 2017-04-10

**Authors:** Tobias Roth, Elias Bader, Patrick Frara, Lorenz Heer, Heinz Flück, Thomas Lüthi, Barbara Schlup, Thomas Schwaller

**Affiliations:** ^1^ University of BaselZoological Institute, Basel Switzerland; ^2^ Hintermann & Weber AGReinachSwitzerland; ^3^ BirdLife SolothurnHägendorfSwitzerland

**Keywords:** delayed plumage maturation, missing data, molt‐constraint hypothesis, morphological monitoring

## Abstract

Individual variation in postjuvenile molt in male Black Redstart is pronounced with about 90% of young males retaining female‐like coloration (*cairei* plumage type) and about 10% acquiring adult male‐like feathers (*paradoxus* plumage type). We examined whether autumn migration timing and body condition differed between individuals of the two plumage types. We used the data of 10,977 Black Redstarts captured during autumn at a ringing site in northern Switzerland where a protocol to record plumage types of captures has been applied since 1980. As *cairei* individuals cannot be distinguished from young females while sexing is comparatively easy for *paradoxus* individuals, the proportion of missing data on sex was likely to be higher for *cairei* individuals than for *paradoxus* individuals. We formally accounted for captures with unidentified sex using a Bayesian approach and conducted a simulation study to show that our approach was able to provide unbiased results even if the proportion of unsexed captures was high. Applying the method to the Black Redstart data, we found that the proportion of individuals with *paradoxus* plumage type increased from 7.6% in 1980 to 18.1% in 2013. Individuals with the *paradoxus* plumage type were on average 0.25 g heavier and had 0.62 mm longer third primaries than individuals with the *cairei* plumage type. However, we found no support for our expectation of later migration of *paradoxus* males compared to *cairei* individuals based on the assumption that *paradoxus* individuals should occupy autumn territories like adult males. Our results shed new light on the understudied timing of autumn migration in birds and are in line with available studies on Black Redstarts, suggesting a molt‐constraint that allows only young males in good body condition to molt into adult‐like plumages.

## 1 Introduction

Most songbirds perform a partial molt shortly after fledging during summer or early autumn (postjuvenile molt; Jenni & Winkler, 1994). Thus, the plumage that an individual will carry during its first breeding season will be acquired only a short time after fledging (Crates, Sheldon, & Garroway, 2015). If plumage traits affect breeding performance (Andersson, 1994), any factor that will have an effect on the extent on postjuvenile molt may also have consequences on how an individual will perform during breeding in the following year. A better understanding of this carry‐over effect could be especially important to enhance our understanding why in some bird species, parts of the individuals do not acquire the definitive plumage during postjuvenile molt, a phenomenon known as delayed plumage maturation (Hawkins, Hill, & Mercadante, 2012).

The extent of the postjuvenile molt, and therefore the proportion of adult‐like feathers in the first breeding plumage, often varies greatly between individuals (Crates et al., 2015; Gargallo, 2013). In male Black Redstarts *Phoenicurus ochruros,* a short‐distance migrant in central Europe, the individual variation in coloration after postjuvenile molt is pronounced but comparatively constant among populations: About 10% of young (i.e., first‐year) males acquire an adult male‐like plumage during postjuvenile molt (*paradoxus* plumage type), while most of young males molt into a dull female‐like plumage (*cairei* plumage type; von Blotzheim, Bauer, & Huber, 1985; Landmann & Kollinsky, 1995a; Nicolai, Schmidt, & Schmidt, 1996). Surprisingly, available studies find little support that the dull coloration of young male Black Redstarts is of any adaptive value (Hawkins et al., 2012; Landmann & Kollinsky, 1995a; Schwarzova, 2010; Weggler, 2001). This is in contrast to most other passerines performing delayed plumage maturation, where dull individuals are less often attacked by adult males, which increases their survival and ultimately enhances their lifetime reproductive success (Hawkins et al., 2012).

Most central European Black Redstarts leave their breeding areas in October for wintering in the Mediterranean (Landmann, 1996), and only few individuals spend the whole year close to their breeding grounds (Maumary, Vallotton, & Knaus, 2007). While most of the population studies on Black Redstart have been conducted during breeding or wintering (Cuadrado, 1995a,b; Landmann & Kollinsky, 1995a,b; Schwarzova, Stros, Frynta, & Fuchs, 2010; Weggler, 2006), only few studies have investigated Black Redstarts in autumn shortly after postjuvenile molt. A particular exception is the field study by Weggler (2000) that examined autumnal singing in Black Redstarts: It is mostly adult male Black Redstarts (potentially including *paradoxus* individuals) past their first breeding season that are engaged in autumnal singing, which allows them to secure access to territories and high‐quality females for the following breeding season. Weggler (2000) concluded that the autumn territoriality of adult birds could explain why young birds are not able to compete with adults for limited breeding opportunities and thus may play a key role for the evolution of delayed plumage maturation (Weggler, 2000).

We here aim to add to the knowledge of autumnal behavior in Black Redstart by analyzing data from a ringing site in northern Switzerland where the plumage types of 10,977 captured Black Redstarts were noted during autumn migration since 1980. While an adult‐like plumage might be beneficial for all young males, part of the young males might be developmentally constrained from achieving it (Hawkins et al., 2012). Such a constraint could arise because body condition does not allow all individuals to invest the resources needed for feather production (Rohwer & Butcher, 1988). We therefore examined whether body condition—measured by the length of the third primary and by body weight—differs between individuals of the two plumage types. Further, we expect that *paradoxus* individuals should invest comparatively more into their first breeding season than *cairei* individuals because the formers are preferred by females (Landmann & Kollinsky, 1995a). As males that defend autumn territories benefit from mating with experienced females the next breeding season (Weggler, 2000), we predict that *paradoxus* males should occupy autumn territories and thus migrate later than *cairei* individuals. We therefore compare migration timing between *paradoxus* and *cairei* individuals.

We acknowledge that our study will not be able to resolve whether delayed plumage maturation has an adaptive function in Black Redstart, as there are no fitness measures of captured individuals available. However, our results shed light on the currently understudied timing of autumn migration in birds (Jenni & Kéry, 2003). Further note that to analyze the ringing data of Black Redstart, we have to use a statistical framework that is able to account for captures with unknown sex. This seems important because it is impossible to identify the sex of most of the *cairei* individuals, while sexing of *paradoxus* males is comparatively easy (Jenni & Winkler, 1994; Nicolai et al., 1996). Simply removing the captures with missing sex from the analyses would result in biased results because the proportion of captures with missing sex likely depended on the plumage type.

Bayesian inference treats all types of unknown quantities including missing values as random variables and, consequently, it provides a formal mechanism to account for missing data, including cases of missing data in discrete variables such as sex (Gelman & Hill, 2007; Kéry & Schaub, 2011; Link & Barker, 2010). We start with describing a statistical model that uses the length of the third primary, the weight and the date of capture to assign the sex or age of captures with missing data. We perform a simulation study to show that our approach is able to reduce the bias of parameter estimates in cases where sex or age was not identified for all individuals and when the proportion of unidentified sex varied between age‐classes. We then use the statistical model to select only the data of young males to investigate differences in *cairei* and *paradoxus* individuals and discuss the results in relation to delayed plumage maturation.

## 2 Materials and Methods

### 2.1 Bird ringing at Subigerberg

Since 1968, birds were regularly caught and ringed at Subigerberg (47°15′N, 7°26′E, elevation 964 m). The ringing station is located in the Jura Mountains in northern Switzerland. Each year during autumn, migration birds are caught with mist nets. The position and length of the nets remained essentially unchanged between years. The average starting day of the ringing period was 23 September; the last day of the ringing period was on average 24 October. On average ±*SD*, the ringing period lasted 28 ± 2.1 days. Within this period, ringing took place on all days except for days with heavy rain or heavy wind. Ringing was performed according to the guidelines of the Swiss Ornithological Institute Sempach; measurement of the third primary was taken according to Jenni and Winkler (1989).

In 1980, a protocol was implemented to describe the plumage coloration of Black Redstarts *Phoenicurus ochruros*. According to this protocol, all captured Black Redstarts aged as first‐year individuals (recognizable by molt limit within greater coverts and tertials; von Blotzheim et al., 1985; Jenni & Winkler, 1994) with signs of adult male plumage were assigned to one of 20 different plumage types (Appendix S1 describes the 20 plumage types). The different plumage types are defined by combinations of the presence of black body feathers, presence of black throat feathers, presence of white mirror on secondaries/tertials, and the combination of these characters. For the present study, we used “*paradoxus*” to refer to young (i.e., first‐year) males that carried one of these 20 plumage types that represent intermediates to adult male plumage, while we used “*cairei*” to refer to young males with female‐like plumage (Nicolai et al., 1996). Note, however, that the sex of young individuals that are female‐colored cannot safely be identified (Nicolai et al., 1996). Consequently, in the ringing data, the plumage of all female‐like individuals was labeled “female‐colored” which included males and females.

We analyzed the data of all first captures of Black Redstarts that were captured between 1980 and 2013 and for which the date of capture, the weight, and the third primary length were available. This resulted in a total of 10,977 individuals. For these individuals, age was missing for 7.3% of the captures and sex was missing for 80.7% of captures. On average, 323 (*SD* = 105) Black Redstarts were ringed per year.

### 2.2 Statistical model

For simplicity, we only considered birds that were captured for the first time. The full data table for the i=1,…,N captured individuals contained the capture day (Day_*i*_, with ${\text{Day}}_{i}=0$ for the day in the middle of the capturing period), year of capture (${\text{Year}}_{i}$), age (${\text{Age}}_{i}=0$ for young individuals; ${\text{Age}}_{i}=1$ for adult individuals; ${\text{Age}}_{i}=\text{NA}$ for individuals with unidentified age), sex (${\text{Sex}}_{i}=0$ for females; ${\text{Sex}}_{i}=1$ for males; ${\text{Sex}}_{i}=\text{NA}$ for individuals with unidentified sex), body weight (${\text{Weight}}_{i}$), and third primary length (${\text{Primary}}_{i}$). Note that we use a Bayesian approach based on Markov chain Monte Carlo (MCMC) to analyze our model (see below). MCMC explicitly imputes missing values at each iteration of the MCMC chain (Gelman & Hill, 2007). Therefore, we can describe the statistical model as if there were no missing values, and MCMC will then correctly handle uncertainty in parameter estimates due to missing values.

We distinguished four groups of Black Redstarts, which were young females (g=1), young males (g=2), adult females (g=3), and adult males (g=4). We used the term “age‐classes” to refer to these four groups. We assumed that the values for body weight and primary length were samples from a multivariate normal distribution expressed as

$$\left(\begin{array}{c} {\text{Weight}}_{i}\\ {\text{Primary}}_{i} \end{array}\right)∼\text{MVN}\left(\left(\begin{array}{c} w0_{g}+w1*{\text{Day}}_{i}+w2*{\text{Year}}_{i}\\ p0_{g}+p1*{\text{Day}}_{i}+p2*{\text{Year}}_{i} \end{array}\right),\left(\begin{array}{cc} \sigma {\text{Weight}}_{g}^{2} & \rho \sigma {\text{Weight}}_{g}\sigma {\text{Primary}}_{g}\\ \rho \sigma {\text{Weight}}_{g}\sigma {\text{Primary}}_{g} & \sigma {\text{Primary}}_{g}^{2} \end{array}\right)\right),$$

where the first parameter of the multivariate normal distribution (MVN) is a vector with the expected body weight and primary length, and the second parameter is the covariance matrix. The expected body weight is given by the age‐class‐specific intercept $w0_{g}$, the linear change of body weight of captured individuals over the season w1, and the linear change of body weight over the study years w2. Similarly, the expected primary length is given by the age‐class‐specific intercept $p0_{g}$, the linear change of primary length of captured individuals over the season p1, and the linear change of primary length over the study years p2. The covariance matrix of the multivariate normal distribution is given by the standard deviation of body weights between individuals of the same age‐class $\sigma {\text{Weight}}_{g}$, the standard deviation of primary length between individuals of the same age‐class $\sigma {\text{Primary}}_{g}$, and the correlation ρ between body weight and primary length.

We further assumed that the probability that a captured individual was a male or that a captured individual was adult may change over the season or over the study years and these changes can be described with logistic regressions:

$$\begin{array}{cc} {\text{Sex}}_{i} & ∼\text{Bernoulli}\left(\text{inv‐logit}\left(s0+s1*{\text{Day}}_{i}+s2*{\text{Year}}_{i}\right)\right)\\ {\text{Age}}_{i} & ∼\text{Bernoulli}\left(\text{inv‐logit}\left(a0+a1*{\text{Day}}_{i}+a2*{\text{Year}}_{i}\right)\right) \end{array}$$

Thus, whether a captured individual was a female or a male is a realization of a Bernoulli distribution and the probability that an individual is a male is described by the logit‐linear predictor with intercept *s*0, the linear change over the season *s*1, and the linear change over the study years *s*2. Similarly, whether a captured individual was young or adult is a realization of a Bernoulli distribution and the probability that an individual was adult is described by the logit‐linear predictor with intercept *a*0, the linear change over the season *a*1, and the linear change over the study years *a*2.

### 2.3 Simulation study

We conducted a simulation study to examine whether we would be able to obtain reasonable results from applying the above‐described statistical model when there were missing values for sex or age. The simulation study was designed to mirror the Black Redstart data from Subigerberg. Accordingly, we simulated a ringing campaign that lasted for 30 years, and on average, 300 individuals were captured each year during a capturing period of 20 days. The capturing period was fixed over the years and only covered part of the species’ migration period. We assumed three different scenarios with differences in the amount of captures with missing age and sex identification.

For the first scenario, we assumed that the sex of a large proportion of young individuals was not identified (young females: 90% with missing sex, young males: 80% with missing sex), while the proportion of sexed individuals was larger for adults (adult females: 30% with missing sex, adult males: 10% with missing sex). In contrast, the proportion of captures with missing age was assumed to be smaller: 40% of young females with missing age, 40% of young males with missing age, 40% of adult females with missing age, and 10% of adult males with missing age. For the second scenario, we assumed that none of the young captures could be sexed (i.e., 100% of young females and young males with missing sex), while all the other settings were the same as in the first simulation scenario. Finally, for the third simulation scenario, we assumed that average body weight and average primary length were the same for the four age‐classes, while all the other settings were the same as in the first simulation scenario.

In Appendix S2, we describe the simulation study in detail including R code to simulate the data for each scenario. For each scenario, we simulated 100 data sets. We analyzed each simulated data set using a Bayesian approach based on Markov chain Monte Carlo (MCMC) methods (Brooks, Catchpole, Morgan, & Harris, 2002). Details of the MCMC analyses including the description of the vague priors that we used for all parameters are also given in Appendix S2. We used the means of the simulated values of the posterior distributions as point estimates of the parameters and the 2.5% and 97.5% quantiles as estimates of the 95% credible intervals. To summarize the results of the 100 simulations, we used the credible interval coverage, which is the proportion of times the true value is contained in the 95% credible interval, and the bias, which is expressed as the difference between estimated and true values. To show the advantage of our approach compared to traditional analyses, we also applied each simulated data set to linear models (LM). We used primary length or body weight as dependent variable and day of the year and year as predictor variables to infer whether the morphological traits of captures were changing over the season and the study years, respectively. We applied these linear models to all data (LM1) and only to the individuals that were identified as first‐year females (LM2).

### 2.4 Statistical analyses of Black Redstart data

We conducted Bayesian inference based on Markov chain Monte Carlo (MCMC) analyses. To reflect our indifference about the parameter values prior to the analyses of the data, we used weakly informative priors (Gelman et al., 2013): normal distributions with mean = 16 and *SD* = 5 for the expected body weight $w0_{g}$ of each age‐class g, a normal distributions with mean = 65 and *SD* = 5 for the expected primary length $p0_{g}$ of each age‐class *g*, normal distributions with mean = 0 and *SD* = 1 for the linear changes over the season and the study period of body weight (w1,w2) and primary length (p1,p2), an inverse Wishart distribution with scale matrix = $\left[\begin{array}{cc} 10^{-4} & 0\\ 0 & 10^{-4} \end{array}\right]$ and degree of freedom = 3 for the covariance matrix of the multivariate normal distribution, normal distributions with mean = 0 and *SD* = 2 for the logit probability that a captured individual is a male (*s*0) and that a captured individual is adult (*a*0), and normal distributions with mean = 0 and *SD* = 1 for the linear changes over the season and the study period for the probability that an individual is a male (s1,s2) and that an individual is adult (a1,a2). MCMC analyses were conducted using JAGS 3.3.0 (Plummer, 2003) and were executed in R using the R add‐on library rjags. Posteriors were based on two parallel chains with 6000 iterations each, discarding the first 2000 values and thinning the remainder by using every second value. Thus, inference was based on 4000 iterations from the posterior distribution. We assessed convergence using trace plots and the Gelman–Rubin diagnostic (Brooks & Gelman, 1998). Further, we used the means of the simulated values of the posterior distributions as point estimates of the parameters and the 2.5% and 97.5% quantiles as estimates of the credible intervals.

Based on the MCMC analyses, we got simulated values from the posterior distribution of the sex and age for all captured Black Redstarts that could not be sexed and/or aged. We used these posterior simulations to select the young males from the data set. To infer whether young males of the *paradoxus* plumage differed from young males of the *cairei* plumage in respect to migration timing, we used a linear model with day of year as response variable and year of capture and plumage type (0 = *cairei*; 1 = *paradoxus*) as predictor variables. To analyze plumage type‐specific body condition, we used linear mixed models with body weight or primary length as response variables and day of year, year of capture and plumage type as predictor variables. We further added year as random effect to account for the year‐to‐year variation in body condition. To consider both the uncertainty in sex and age estimates and the uncertainty in parameter estimates in the linear models, we repeated the following steps for all 4,000 iterations of the posterior distribution from the MCMC analyses: (1) Use the MCMC results to select the young males from the data set, (2) apply the linear mixed models to the data of the selected individuals using the R‐function *lmer*, and (3) get a single estimate from the posterior distribution of parameters in the linear models using the R‐function *sim* in the package *arm*. We thus got 4,000 simulated values of the posterior distribution that accounted for the uncertainty in sex and age estimates as well as in the estimates of plumage‐type effects.

As Black Redstart shifted migration to later dates over the years (Section 3) whereas the yearly capturing window was held constant, the capturing period shifted to cover a relatively earlier window of the migration period of Black Redstarts with increasing study years. To take this shifting capturing window into account, we corrected the capturing day of each captured individual by subtracting 0.2 days per year (Section 3): Thus, an individual captured on 10 October in 2000 would correspond to an individual captured on the 6 October in 1980. However, results based on the corrected capturing day did not change quantitatively from the results with uncorrected capturing days. We therefore present only the results based on the uncorrected capturing days.

## 3 Results

### 3.1 Simulation results

Detailed results of the simulation exercise using the three simulation scenarios are given in Appendix S3. Generally, the results of the three simulation scenarios suggest that our statistical approach was able to accurately estimate model parameters. In our three simulation scenarios, credible interval coverage was close to the nominal level of 95% and bias was generally low. In contrast, the performance of the two traditional linear models was weak. When all data without accounting for sex and age were used, the results of the linear model were strongly biased and for instance suggested that the average primary length of captures increased over the years (simulation setting: no change over the years). This bias is due to the shift in phenology of captured individuals toward later dates over the course of the study. As the capturing period was fixed over the years, the proportion of adults and the proportion of males captured during the study period both increased over the years and, consequently, individual captures apparently became larger over the study years. In contrast, when only one age‐class was used, and therefore captures with missing sex or age were removed, the results were not bias but the precision of the estimated trend over the year was much lower as compared to our statistical model. This low precision of estimates in the linear model is because of reduced sample size due to the removal of captures with missing sex or age.

### 3.2 Autumn migration of Black Redstarts at Subigerberg

The total number of Black Redstarts captured per season slightly but nonsignificantly decreased by 3.0 (95% credible interval: −6.6 to 0.7) captures per year. Although the capturing period was held constant over the study years, the average date of capture of all Black Redstart that were captured per year increased about 0.20 [95% credible interval: 0.19 to 0.22] days per year, implying that Black Redstarts in 1980 migrated on average about a week earlier than in 2013.

**Figure 1:**
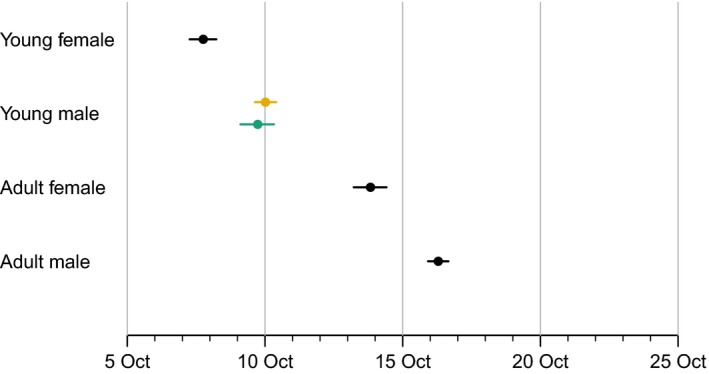
Differences in average day of capture between the age‐classes. Given are mean and 95% credible intervals of the posterior distribution. For young males, the average day of capture is given for individuals carrying the cairei plumage type (orange bar) and for individuals carrying the paradoxus plumage type (green bar). The sex and age of captures with missing data were estimated based on the described statistical model (Section 2)

The probability that a captured individual was a male decreased over the study period from 1980 to 2013 (*s*2: −0.046 [95% credible interval: −0.056 to −0.035]), while the probability that a captured individual was adult increased over the study period (*a*2: 0.017 [0.010 to 0.024]). These results only marginally changed if we used capturing dates that were corrected for the increasingly later migration of Black Redstarts. Within a migration season, the probability that a captured individual was a male increased over the season (*s*1: 0.032 [0.022 to 0.043]). Similarly, the probability that a captured individual was an adult individual increased over the season (*a*1: 0.122 [0.113 to 0.131]). Consequently, average date of capturing within the capturing period was earliest for young females and latest for adult males (Figure 1).

Among the young males, the average percentage of individuals with *paradoxus* plumage was in average 11.3% (range of estimates due to uncertainty in the selection of young males from captures with missing sex/age: 10.6% to 12.1%). The percentage of young males with *paradoxus* plumage increased from 7.6% (95% credible interval: 6.4% to 8.9%) in 1980 to 18.1% (95% credible interval: 15.4% to 20.9%) in 2013. The probability that an increase in the proportion of *paradoxus* individuals is supported based on our data and the described statistical model was >0.99. We expected that *paradoxus* individuals would more closely mimic adult male behavior than *cairei* individuals and thus predicted later passage of *paradoxus* individuals within a season. However, the probability that this prediction is supported based on our data and the described statistical model was only 0.22 and the average migration date was estimated at 10 October for both *cairei* and *paradoxus* individuals (Figure 1).

**Figure 2:**
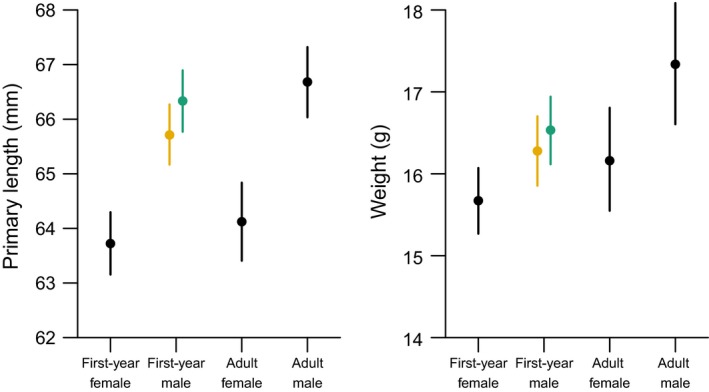
Differences in average length of third primary (left panel) and average body weight (right panel) among the age‐classes. Given are mean and 95% credible intervals of the posterior distribution. For young males, numbers are given for individuals carrying the cairei plumage type (orange bar) and individuals carrying the paradoxus plumage type (green bar). The sex and age of captures with missing data were estimated based on the described statistical model (Section 2)

Using our statistical approach, we found that both the body weight (*w*2: 0.011 [0.008 to 0.014]) and the primary length (*p*2: 0.015 [0.010 to 0.020]) of captures increased between 1980 and 2013. Within a migration season, the primary length decreased over the season (*p*1: −0.014 [−0.020 to −0.008]), while the average weight increased over the season (*w*1: 0.027 [0.024 to 0.030]). Note that, when using traditional linear models without accounting for the sex‐age‐classes, the conclusion for primary length would be different: Primary length of captured individuals apparently increases over the season (LM; slope = 0.01 [0.01 to 0.02]) because the proportion of captured males increased over the season and the primary length of males is larger than the primary length of females (Figure 2). Finally, young males carrying the *paradoxus* plumage type were on average 0.25 [0.16 to 0.36] g heavier and had 0.62 [0.47 to 0.78] mm longer primaries than males carrying the *cairei* plumage type. Based on the data and the statistical model, the probability that *paradoxus* individuals were longer‐winged and heavier was 0.95 and 0.81, respectively.

## 4 Discussion

Data from bird ringing schemes provide long‐running ecological time series. In combination with appropriate statistical methods that account for missing values, these data are important sources to document the temporal change of bird populations under global change (Jenni & Kéry, 2003; Thorup, Korner‐Nievergelt, Cohen, & Baillie, 2014) and provide insight into the processes leading to intraspecific variation in species (Telleria, de la Hera, & Perez‐Tris, 2013). In this study, we used a statistical model to describe the differences in young/adult and male/female Black Redstarts according to body weight, primary length, and migration timing and used that model to assign the sex of captures with unidentified sex or age. Using simulations, we show that applying our approach outperforms traditional approaches based on linear models that either use all data without accounting for sex or age, resulting in biased results, or approaches that use only the data with identified sex and age, resulting in less precise results. Our approach thus makes efficient use of available data.

Applying our method to the data of 10,977 Black Redstarts that were captured from 1980 to 2013 during autumn migration at a ringing station in northern Switzerland, we found that young males with adult male‐type feathers (*paradoxus* plumage type) were 0.25 g heavier and had 0.62 mm longer third primaries than young males carrying the dull (*cairei*) plumage type. As it is likely that these differences in body condition reflected differences in male quality for instance because mass gain after fledging depends on foraging efficiency (Wunderle, 1991), our results suggest that females selecting adult‐like males at the breeding sites will select among the young males the ones that had higher quality during the year of fledging.

Earlier spring arrival of males compared to females can be explained by a trade‐off between reproductive benefits of territory possession and survival costs of arriving too early in the season (Kokko, 1999; Kokko, Gunnarsson, Morrell, & Gill, 2006). Similar trade‐offs could result in sex‐specific autumn migration timing (Mills, 2005). While adult male Black Redstarts that occupy territories in autumn between the end of molt and the start of migration profit from mating with experienced females the following breeding season, young female‐like males rarely defend territories in autumn (Weggler, 2000). Thus, the benefits of autumnal territory possession for adults would predict later autumn migration of adults, which matches well with our results (Figure 1). Note that the benefit of wintering territories is less clear: In southern Spain, about half of adult males seem to defend stable winter territories, about half of the adult males and some of the female‐colored birds defend several territories throughout the winter, while most of the female‐colored birds are nonterritorial (Cuadrado, 1995b).

For the young males of the *paradoxus* plumage type, we predicted later migration compared to individuals of the *cairei* type, because *paradoxus* males are investing relatively more energy to produce the costlier blackish feathers and thus seem to invest relatively more into their first breeding season than males of the *cairei* plumage type. Consequently, we expected that *paradoxus* males should use the period of autumn singing to occupy their own territories or to acquire information about occupied territories (Pinowski, Vaclav, Pinowska, & Romanowski, 2014). Such information could benefit *paradoxus* males to make appropriate decision where to settle in the following season. Our results, however, do not seem to support this reasoning as we found that *paradoxus* males migrated in average at the same time as *cairei* individuals. Similar to Weggler (2000), we lack a conclusive explanation why young males particularly of the *paradoxus* type migrate early and do not seem to take part in contests for territories in autumn.

The breeding populations of Black Redstarts in Europe moderately increased in Europe since 1980 (BirdLife International 2016). This increase in breeding population, however, was not reflected in the numbers of captured Black Redstart at the ringing station that even showed a slight decrease over the study period presumably because an increasing proportion of individuals seem to migrate after the capturing period or even entirely skipped migration. Yet, the body weight and primary length increased over the study period between 1980 and 2013, suggesting that the overall favorable conditions that seem to promote population increase in Europe also led to better body condition of captured Black Redstarts. As shorter wings are usually associated with shorter migration distances (Telleria et al., 2013), increased primary length of captures over the study period could also be explained by different population structure; that is, short‐distance populations of Black Redstarts skip migration, while long‐distance migrants did not adapt migration behavior. Clearly, to better understand the temporal change in body condition and migration timing of captures at the Subigerberg, we would need more information about the breeding origins of captures, which might be available soon due to advances in bird tracking techniques (Bridge et al., 2011; Lopez‐Lopez, 2016).

While individuals of the *paradoxus* plumage type show attributes of adult male plumage, we found little support of adult male‐like behavior in *paradoxus* individuals during autumn migration: While adult males migrated considerably later than adult females, *paradoxus* males did not migrate later during autumn migration than *cairei* individuals. Furthermore, while the probability to capture males decreased over the study years, suggesting that males were increasingly more likely to remain at the breeding sites, the proportion of *paradoxus* individuals among young males even increased over the study period. Taken together, we conclude that while heavier and longer‐winged young males were more likely to carry adult male‐like plumages, these males do not yet behave like adult males during autumn migration.

## Conflict of Interest

The authors declare that they have no competing interests.
