# Supplementary material for: Timing and body condition of dichromatic Black Redstarts during autumn migration
Source: Ecol Evol. 2017 Apr 10;7(10):3567–73. doi: 10.1002/ece3.2911 (PMC5433976; doi:10.1002/ece3.2911)
Supplement: Supplementary file 1 [file ECE3-7-3567-s001.docx]

Appendix S1. Description of the 20 Plumage Types of 1st Year Males With Adult Male-like Feathers

At the ringing station 'Subigerberg' in 1980 a protocol was implemented to describe the plumage colouration of Black Redstarts (*Phoenicurus ochruros*). According to this protocol first-year individuals with signs of adult male plumage were assigned to one of the following plumage types:

| Type-ID | Description |
| --- | --- |
| 2 | no white mirror, body feathers as adult male (i.e. the plumage that is usually described under the name *paradoxus* in the literature) |
| 1f | no white mirror, only few blackish body feathers |
| la | white mirror on S7-9, body feathers similar to female |
| lb | white mirror on S7-8, body feathers similar to female |
| lc | white mirror on S8-9, body feathers similar to female |
| ld | white mirror only on S8 or on S9, body feathers similar to female |
| le | white mirror on S6-7; black throat |
| lf | white mirror on S5-8; body feathers as adult male |
| lg | white mirror on S7-8, body feathers similar to female |
| lh | white mirror on S7-8; body feathers as adult male |
| li | white mirror on S6; black throat |
| lk | white mirror on S7; body feathers as adult male |
| ll | white mirror on S8; body feathers as adult male |
| lm | white mirror on S7, body feathers similar to female |
| ln | white mirror on S5-6, body feathers similar to female |
| lo | white mirror on S9, body feathers similar to female |
| lp | white mirror on S8-9; body feathers as adult male |
| lq | white mirror on S9; body feathers as adult male |
| lr | white mirror on S6-9, body feathers intermediate between female and adult male |
| ls | white mirror on S7-9, body feathers similar to female |

S: Secondaries (Tertials: S7-9)
