# Supplementary material for: Timing and body condition of dichromatic Black Redstarts during autumn migration
Source: Ecol Evol. 2017 Apr 10;7(10):3567–73. doi: 10.1002/ece3.2911 (PMC5433976; doi:10.1002/ece3.2911)
Supplement: Supplementary file 2 [file ECE3-7-3567-s002.docx]

Appendix S2. Description of Simulation Study

Preamble

Appendix S2 describes (1) how we simulated the data that have a similar structure as the ringing data of Black Redstarts (*Phoenicurus ochruros*) from the Subigerberg, (2) the three simulation scenarios considered in the simulation study, (3) the statistical model in bugs language, and (4) the MCMC settings to analyse one set of simulated data. Throughout, R or BUGS code is given in consolas font.

The following R-libraries are needed for the simulation and the analyses of the simulated data using JAGS (Plummer 2003).

# Libraries
library(MCMCpack) # needed to simulate from wishard distri.
library(rjags) # needed to run JAGS form R

Description of Data Simulation

In the following we describe the main ideas of the simulation and our standard setting that we used for the entire simulation study. For each scenario we slightly changed some the standard setting to infer how this will affect the accuracy of parameter estimates under our method.

Setting of the Simulation

The simulation procedure had three main steps:

1. *Describe the phenology of captured individuals independent of sex and age*: First we simulated the number of captured individuals per year and assigned when (i.e. the Julian day) each individual was captured. We used the following settings to describe the distribution of captures over years and season:

Nyear <- 30 # number of study years
Ncap <- 300 # average number of captures per year
Nperyear <- rpois(Nyear, Ncap) # number of captures per year
arrival <- 0 # mean arrival at year 0
trend.arrival <- 0.2 # trend over years in mean arrival
arrival.sd <- 5 # SD of individual arrival
start <- -10 # Start of study period (Julian day)
end <- 10 # End of study period (Julian day)

1. *Assign individual characters to each captured individual:* Given the year and Julian day an individual was captured (as described in the first part of the simulation), we assigned its age, sex, weight and primary length. To define the average weight and primary length per age class (i.e. first year females, first year males, adult females and adult males), we used a vector with the first element for the average of first year females, the second element for the average of first year males, the third element for the average of adult females and the fourth element for the average of adult males. For the simulation we generally assumed that a captured individual was more likely to be a first year female early in the season and was more likely to be an adult male late in the season (i.e. s1>0 and a1>0; see below, and formulas in the main body of the manuscript). Further we assumed that there were slight differences in average weight and average primary length between the four age classes. Further, we assumed that the weight and the primary length of individuals were positively correlated. We used the following settings to assign the characters of the captured individuals:

s0 <- logit(0.3) # logit prob. that a captured ind at day
 # 0 and year 0 is a male
s1 <- 0.1 # change over the season
s2 <- 0 # change over the years
a0 <- logit(0.1) # logit prob. that a captured ind at day
 # 0 and year 0 is adult
a1 <- 0.1 # change over the season
a2 <- 0 # change over the years
p0 <- c(65.0, 66.4, 64.3, 67.0) # avg. primary length of an ind
p0.sd <- c(1.5, 1.5, 1.6, 1.6) # SD of primary length between ind
p1 <- 0 # change avg. primary length over season
p2 <- 0 # change avg. primary length over years
w0 <- c(14.5, 16.5, 16.4, 17.5) # avg. weight of an ind
w0.sd <- c(1.2, 1.2, 1.3, 1.4) # SD of weight between ind
w1 <- 0 # change of avg. weight over season
w2 <- 0 # change of avg. weight over years
rho <- 0.3 # correlation of weight and primary len

1. *Simulate unidentified individuals*: Finally, we changed the sex and/or age for some captured individuals to ‘not identified’ (i.e. NA). For each group of individuals (i.e. first year females, first year males, adult females and adult males) we randomly assign NA values to sex and age according to the following settings:

# Probability that the sex of an ind is not identified
nonID_Sex <- c(0.9, 0.8, 0.3, 0.1)

# Probability that the age of an ind is not identified
nonID_Age <- c(0.4, 0.4, 0.4, 0.1)

Simulation function

The following function simulates data based on the three steps and settings as described above:

simdat <- function() {
 # 1. Distribute captures over years and season
 dat <- data.frame(Year = rep(1:Nyear, Nperyear))
 dat$Julian = floor(rnorm(nrow(dat), arrival + trend.arrival * dat$Year, arrival.sd))
 dat <- dat[dat$Julian >= start & dat$Julian < end,]

 # 2. Assign individual characters
 dat$Sex <- rbinom(nrow(dat), 1, plogis(s0 + dat$Julian * s1 + dat$Year * s2))
 dat$Age <- rbinom(nrow(dat), 1, plogis(a0 + dat$Julian * a1 + dat$Year * a2))
 dat$Group <- 1
 dat$Group[dat$Age==0 & dat$Sex == 1] <- 2
 dat$Group[dat$Age==1 & dat$Sex == 0] <- 3
 dat$Group[dat$Age==1 & dat$Sex == 1] <- 4
 dat$Wing <- NA
 dat$Weight <- NA
 for(i in 1:nrow(dat)) {
 Sigma <- matrix(c(p0.sd[dat$Group[i]]^2,
 rep(rho*p0.sd[dat$Group[i]]*w0.sd[dat$Group[i]], 2),
 w0.sd[dat$Group[i]]^2), nrow = 2)
 mu <- c(p0[dat$Group[i]] + p1[dat$Group[i]] * dat$Julian[i] + p2[dat$Group[i]] * dat$Year[i],
 w0[dat$Group[i]] + w1[dat$Group[i]] * dat$Julian[i] + w2[dat$Group[i]] * dat$Year[i]
 )
 dat[i, c("Wing", "Weight")] <- round(mvrnorm(1, mu, Sigma),2)
 }
 dat$Day = dat$Julian + abs(min(dat$Julian)) + 1

 # 3. Simulate unidentified individuals (i.e. set Age or Sex to NA)
 for(i in 1:nrow(dat)) {
 dat$Sex[i] <- sample(c(dat$Sex[i],NA), 1, nonID_Sex[dat$Group[i]])
 dat$Age[i] <- sample(c(dat$Age[i],NA), 1, nonID_Age[dat$Group[i]])
 }
 grouptrue <- dat$Group
 dat[is.na(dat$Sex) | is.na(dat$Age), "Group"] <- NA

 # 4. Return simulated data
 list(dat=dat, grouptrue=grouptrue)
}

Run Simulation

We can now apply the function to get simulated data. Fig. 1 gives the phenology of captures over the study years and over the season and Fig. 2 the differences in morphological measures between individuals.

simdat <- simdat()
dat <- simdat$dat


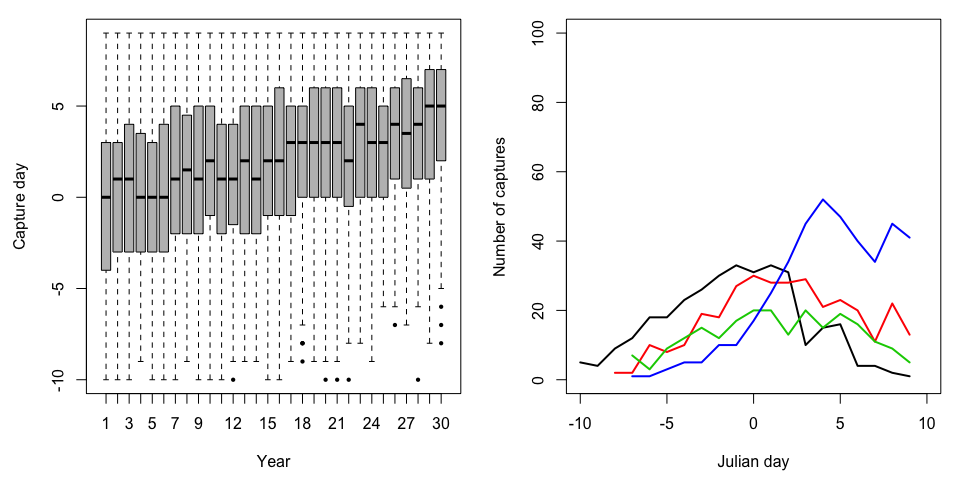


**Fig. 1:** Left panel: boxplots of captured individuals per year . Dates are expressed as Julian days with the middle of the capturing period set as day = 0. The capturing period in all year lasted from the day=-10 to day=10. Right panel: number of captured individuals over the course of the season (all years pooled): given in black are first-year females, in red first-year males, in green adult females and in blue adult males.

**Fig. 2:** Distribution of the primary length and body weights of all individuals. Black dots are first-year females, red dots first-year males, green dots adult females and blue dots adult males. Note that the figure also includes individuals where sex or age will be set as unknown.

Simulation Scenarios

Above we described the general structure of the simulation. For each of three simulation scenario, we slightly changed the settings to infer the performance of our approach under different proportions of captures with unidentified sex or age. For each setting we simulated 100 data sets and estimated the parameters using JAGS as described below. The three scenarios were as following:

Scenario 1: Same Proportion of Missing Values as in Subigerberg Data

The setting was as described above.

Scenario 2: Sex of first-year individuals missing

For this scenario we increased the proportion of missing sex: for all first-year individuals sex could not be identified. We changed the simulation setting as following:

nonID_Sex <- c(1, 1, 0.3, 0.1)

Scenario 3: No morphological differences

For the third simulation scenario we assumed that in average there was no differences in morphological characters (i.e. body weight and primary length) between individuals of the four age classes. We changed the simulation setting as following:

p0 <- c(84.0, 84.0, 84.0, 84.0)
w0 <- c(16.0, 16.0, 16.0, 16.0)

Model description in BUGS language

To estimate model parameters from the simulated data, we used a Bayesian approach based on markov chain monte carlo (MCMC) methods (Brooks 2003). In the following code section the model that was used to estimate parameter values from the simulated data is given in the BUGS language (Link et al. 2002). Since it is a Bayesian analyses the description of the model also contains the priors.

mod <- function() {
 # PRIORS
 # Intercept and temporal trend for avg. primary length
 # - p0[1,1]: intercept for 1st yr females
 # - p0[2,1]: intercept for 1st yr males
 # - p0[1,2]: intercept for adult females
 # - p0[2,2]: intercept for adult males
 p0[1,1] ~ dnorm(65, 0.04)
 p0[1,2] ~ dnorm(65, 0.04)
 p0[2,1] ~ dnorm(65, 0.04)
 p0[2,2] ~ dnorm(65, 0.04)
 p1 ~ dnorm(0, 4) # change in primary length over season
 p2 ~ dnorm(0, 4) # change of primary length over years

 # Intercept and temporal trends for weight
 # - w0[1,1]: intercept for 1st yr females
 # - w0[2,1]: intercept for 1st yr males
 # - w0[1,2]: intercept for adult females
 # - w0[2,2]: intercept for adult males
 w0[1,1] ~ dnorm(16, 0.04)
 w0[1,2] ~ dnorm(16, 0.04)
 w0[2,1] ~ dnorm(16, 0.04)
 w0[2,2] ~ dnorm(16, 0.04)
 w1 ~ dnorm(0, 4) # change in body weight over season
 w2 ~ dnorm(0, 4) # change in body weight over years

 # Average (mu) and covariance matrix (Sigma) of multinormal distribution
 for(t in 1:Nyear) {
 for(d in 1:Ndays) {
 # Primary length
 mu[t,d,1,1,1] <- p0[1,1] + p1*d + p2*t
 mu[t,d,1,2,1] <- p0[2,1] + p1*d + p2*t
 mu[t,d,1,1,2] <- p0[1,2] + p1*d + p2*t
 mu[t,d,1,2,2] <- p0[2,2] + p1*d + p2*t
 # Body weight
 mu[t,d,2,1,1] <- w0[1,1] + w1*d + w2*t
 mu[t,d,2,2,1] <- w0[2,1] + w1*d + w2*t
 mu[t,d,2,1,2] <- w0[1,2] + w1*d + w2*t
 mu[t,d,2,2,2] <- w0[2,2] + w1*d + w2*t
 }
 }
 tau[1:2,1:2] ~ dwish(S3[,],3)
 Sigma[1:2,1:2] <- inverse(tau[,])

 # Intercept and temporal (within yr: a1; between yr: a2) trend for Age
 a0 ~ dnorm(0, 0.4)
 a1 ~ dnorm(0, 1)
 a2 ~ dnorm(0, 1)

 # Intercept and temporal (within yr: s1; between yr: s2) trend for Sex
 s0 ~ dnorm(0, 0.4)
 s1 ~ dnorm(0, 1)
 s2 ~ dnorm(0, 1)


 ## LIKELIHOOD
 for (i in 1:N) {
 Trait[i, 1:2] ~ dmnorm(mu[year[i], day[i], , Sex[i]+1, Age[i]+1], tau[ , ])
 Sex[i] ~ dbern(pi_s[i])
 logit(pi_s[i]) <- s0 + s1 * Julian[i] + s2 * year[i]
 Age[i] ~ dbern(pi_a[i])
 logit(pi_a[i]) <- a0 + a1 * Julian[i] + a2 * year[i]
 }

}

Parameter Estimation Using MCMC Methods

We now have simulated data and the description of the model to analyse these data. Thus, we are ready to run the MCMC analyses which were conducted using JAGS 3.3.0 (Plummer 2003) and were executed in R using the R add-on library rjags. We run two parallel chains with 4000 iterations each, discarding the first 2000 values and thinning the remainder by using every second value. We used the means of the simulated values of the posterior distributions as point estimates of the parameters and the 2.5% and 97.5% quantiles as estimates of the credible intervals. The following code sections describe the different steps needed to run the model. First we need to prepare the data to be used in JAGS.

jagsdat <- list(
 N = as.integer(nrow(dat)),
 Nyear = max(dat$Year) - min(dat$Year) + 1,
 year = dat$Year - (min(dat$Year)-1),
 Julian = dat$Julian,
 day = dat$Day,
 Ndays = max(dat$Day),
 Trait = dat[, c("Wing", "Weight")],

 Age = dat$Age,
 Sex = dat$Sex,
 S3 = matrix(c(1,0,0,1),nrow=2)/10000
)

Then we also need to prepare sensible initial values. In the following the function that we used to generate initial values is given.

inits <- function() {
 # Give initial values for missing age or sex
 tage <- dat$Age
 tage[is.na(dat$Age)] <- 0
 tage[!is.na(dat$Age)] <- NA
 tsex <- dat$Sex
 tsex[is.na(dat$Sex)] <- 0
 tsex[!is.na(dat$Sex)] <- NA

 # Return a list containing all the initial values
 list(
 Age = tage,
 Sex = tsex,
 w0 = matrix(rnorm(4, 16, 2), nrow = 2),
 w1 = rnorm(1, 0, 0.5),
 w2 = rnorm(1, 0, 0.5),
 p0 = matrix(rnorm(4, 65, 2), nrow = 2),
 p1 = rnorm(1, 0, 0.5),
 p2 = rnorm(1, 0, 0.5),
 tau = rwish(3,matrix(c(.02,0,0,.04),nrow=2)),

 a0 = rnorm(1, 0, 0.2),
 a1 = rnorm(1, 0, 0.2),
 a2 = rnorm(1, 0, 0.2),
 s0 = rnorm(1, 0, 0.2),
 s1 = rnorm(1, 0, 0.2),
 s2 = rnorm(1, 0, 0.2)
 )
}

We also need to define the MCMC settings.

t.n.thin <- 2
t.n.chains <- 2
t.n.burnin <- 2000
t.n.iter <- 2000

Finally, we define for which parameters we aim to get simulated values from the posterior distribution and we can run the model.

param <- c("w0", "w1", "w2", "p0", "p1", "p2", "Sigma", "a0", "a1", "a2", "s0", "s1", "s2")
jagres <- jags.model("mod.R", data = jagsdat,
 n.chains = t.n.chains, inits = inits, n.adapt = t.n.burnin)

References

Brooks, S. P. 2003. Bayesian computation: a statistical revolution. Philosophical Transactions of the Royal Society of London Series a-Mathematical Physical and Engineering Sciences 361:2681-2697.

Link, W. A., E. Cam, J. D. Nichols, and E. G. Cooch. 2002. Of BUGS and birds: Markov chain Monte Carlo for hierarchical modeling in wildlife research. Journal of Wildlife Management 66:277-291.

Plummer, M. 2003. JAGS: A program for analysis of Bayesian graphical models using Gibbs sampling. 3rd International Workshop on Distributed Statistical Computing.
