# Supplementary material for: Timing and body condition of dichromatic Black Redstarts during autumn migration
Source: Ecol Evol. 2017 Apr 10;7(10):3567–73. doi: 10.1002/ece3.2911 (PMC5433976; doi:10.1002/ece3.2911)
Supplement: Supplementary file 3 [file ECE3-7-3567-s003.docx]

Appendix S3: Results of Simulation Study

Preamble

Appendix S3 gives the results of the three simulation scenarios. We will use the following measures to summarize the 100 simulations for each scenario:

- *Credible interval coverage*: The proportion of times the true value is contained in the 95% credible interval.
- *Bias*: The average of the differences between estimated and true value.
- *Power*: The proportion of times the 95% credible interval of a trend estimate does not contain 0 (i.e. no trend). Only sensible if a trend different from 0 was used for the simulation.

We compare the results of our approach with the estimates of a linear model with wing size (e.g. measured as the length of the third primary) or body weight as dependent variable and day of year and year as predictor variables. We estimated the parameters once with all captured individuals and not accounting for the sex and age of the captured individuals (i.e. LM1) and once with only the data of the first year females (i.e. LM2).

Scenario 1: No trend over the years

For the first simulation scenario we used the standard setting of the simulation parameters as described in Appendix S2. The main feature of this setting is that the peak phenology of the captured individuals is postponing over the years (i.e. average day of capturing is becoming later over the years), while for the other parameters there is no systematic change over the years. In Fig. 1 the results of the average wing size for the 100 simulated data sets is given.


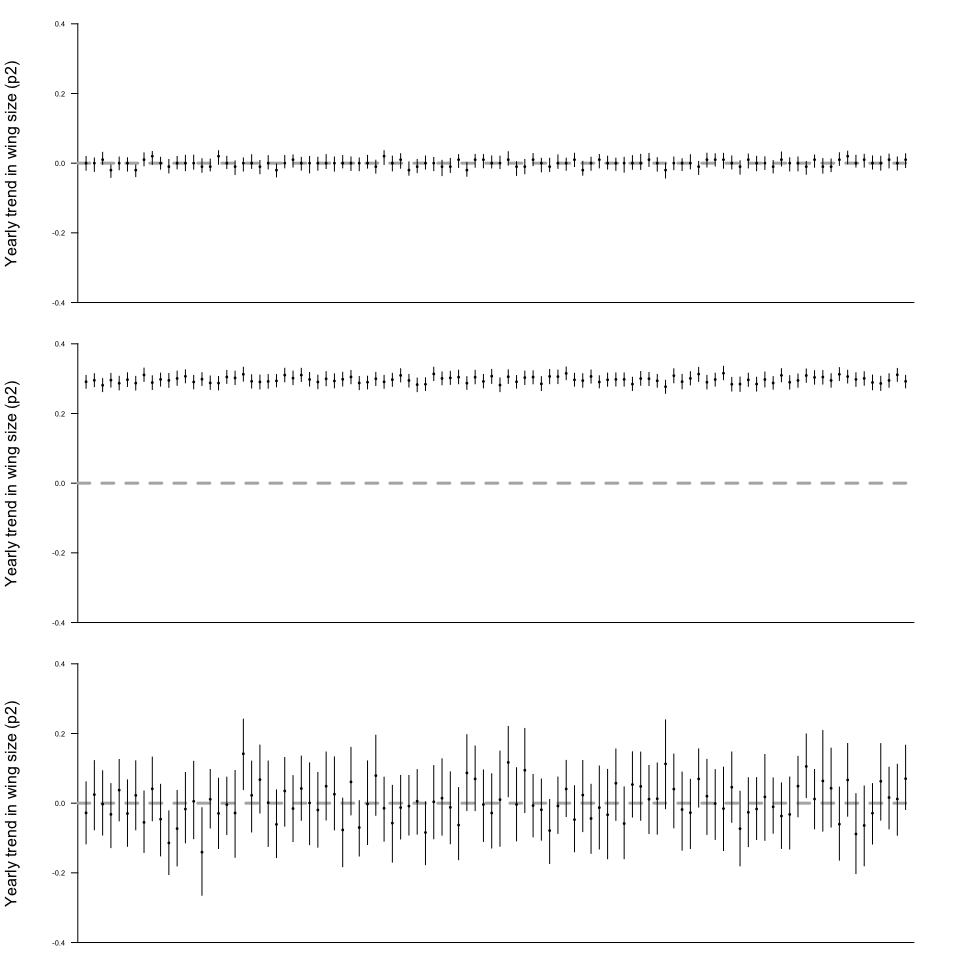


**Figure 1:** Estimated trend of average wing size of first year females from our statistical model (upper panel), LM1 (i.e. all data used without accounting for sex and age; middle panel) and LM2 (i.e. only data of identified first year female captures used; lower panel). Given are for each of the 100 simulations the mean and the 95% credible intervals obtained from the posterior distributions. The dotted line gives the true value assumed in the simulations.

According to the simulation study, our statistical model was able to accurately estimate model parameters. The credible interval coverage, was close to the nominal level of 95%: it was 0.97 for the yearly change in average wing length of first year females and 0.93 for the yearly change in average weight of first year females. Overall, these results suggest that our model provided accurate estimates with reasonable precision under the first scenario. Further, the simulation suggests no bias for the yearly change in average wing length of first year females (bias = 0.00) and average weight of first year females (bias = 0.00).

In contrast, the performance of the two linear models was weak (Fig. 1). When all data without accounting for sex and age were used, the estimates of the linear model suggested that the average wing length of captures increased over the years. This is due to the shift in phenology of captured individuals, the proportion of captured adults increased over the years. In contrast, when only the identified first year females were used in the linear model, the estimate was not biased but the credible intervals were much larger compared to the credible intervals of our statistical approach. This low precision of estimates in the linear model is because the sex and age of only a fraction of the first year females was identified and, thus, only few data points could be used in the linear model.

Scenario 2: Sex of first year individuals unidentified

For the second simulation scenario we assumed that the sex of none of the first year individuals were identified. Thus, using traditional linear (mixed) models one can only use all data (without accounting for sex, i.e. LM1), while LM2 (i.e. using only first year females in the model) is not possible because the sex of first year individuals can not be identified.


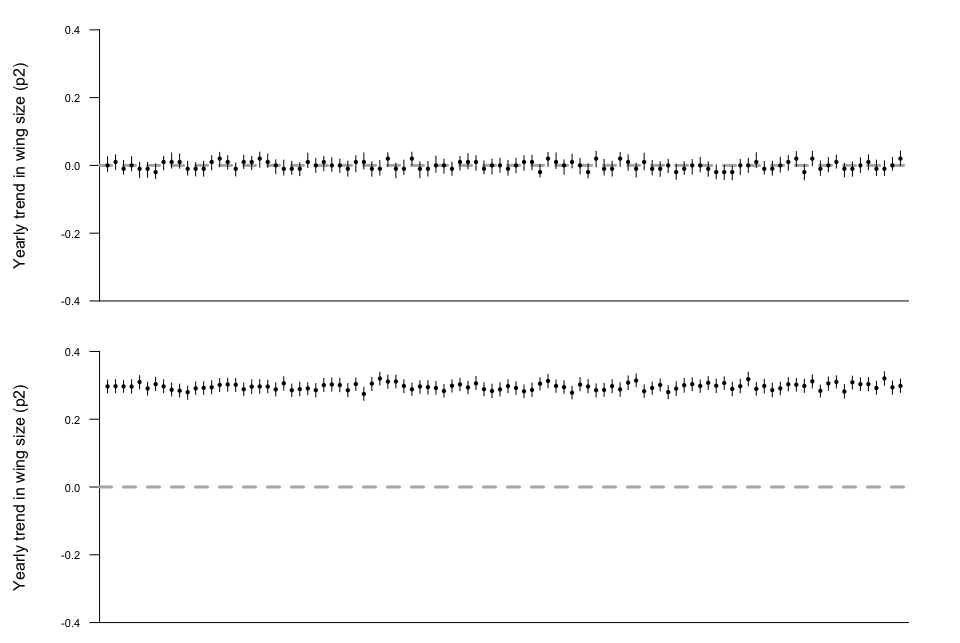


**Fig. 2:** Estimated trend of average wing size of first year females from our statistical model (upper panel) and LM 1 (i.e. all data used without accounting for sex and age; lower panel). Given are for each of the 100 simulations the mean and the 95% credible intervals obtained from the posterior distributions. The dotted line gives the true value assumed in the simulations.

Even though the sex of none of the first year females was identified, our statistical model for morphological monitoring was able to accurately estimate model parameters for first year females. The credible interval coverage, was close to the nominal level of 95%: it was 0.97 for the yearly change in average wing length of first year females and 0.96 for the yearly change in average weight of first year females. Further, the simulation suggests no bias for the yearly change in average wing length of first year females (bias = 0.00) and for the yearly change in average weight of first year females (bias = 0.00).

Similar as in simulation scenario one, when all data without accounting for sex and age were used, the estimates of the linear model suggested that the average wing length of captures increased over the years.

Scenario 3: No morphological differences

For the third simulation scenario we used the standard setting of the simulation parameters as described in Appendix S2. The main feature of this setting is that the peak phenology of the captured individuals is postponing over the years (i.e. average day of capturing is becoming later over the years), while for the other parameters there is no systematic change over the years. In Fig. 4 the results of the average wing size for the 100 simulated data sets is given.


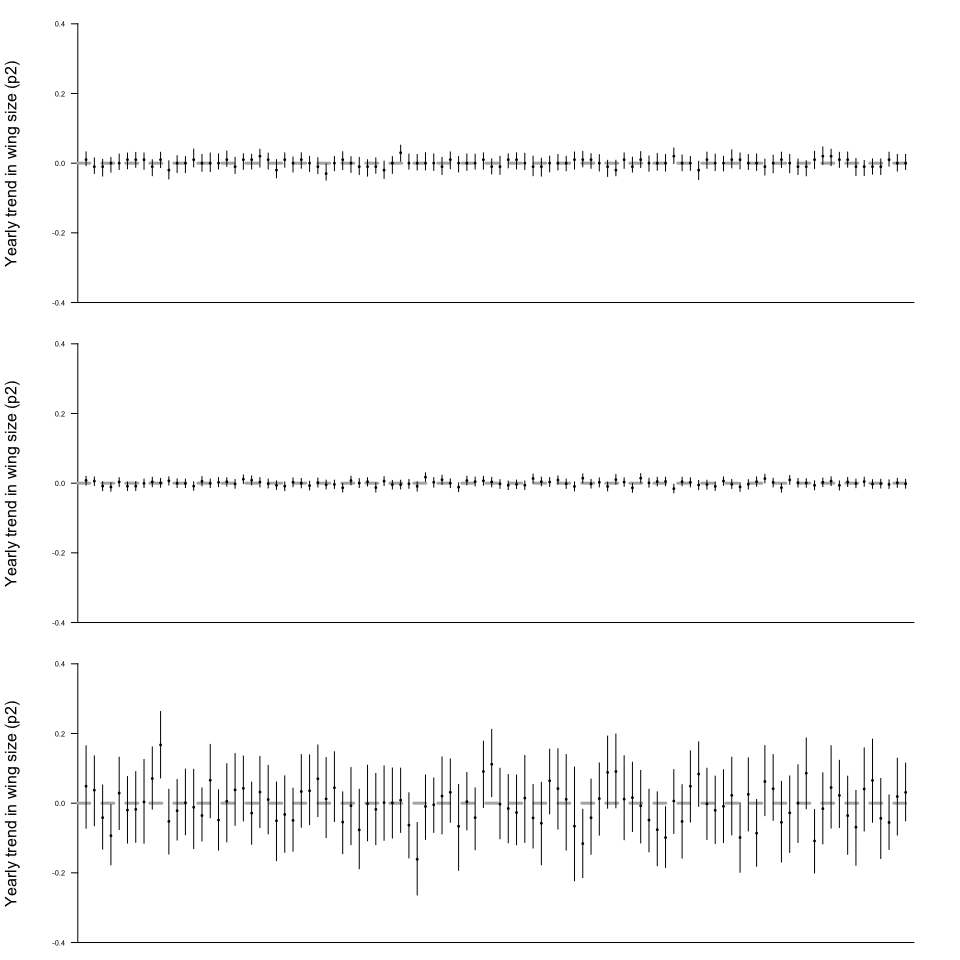


**Fig. 4:** Estimated trend of average wing size of first year females from our statistical model (upper panel), the LM1 (i.e. all data used without accounting for sex and age; middle panel) and the LM2 (i.e. only data of identified first year female captures used; lower panel). Given are for each of the 100 simulations the mean and the 95% credible intervals obtained from the posterior distributions. The dotted line gives the true value assumed in the simulations.

According to the simulation study, our statistical model was able to accurately estimate model parameters. The credible interval coverage was close to the nominal level of 95%: it was 0.98 for the yearly change in average wing length of first year females and 0.91 for the yearly change in average weight of first year females. Overall, these results suggest that our model provided accurate estimates with reasonable precision under the first scenario. Further, the simulation suggests no bias for the yearly change in average wing length of first year females (bias = 0.00) and for the yearly change in average weight of first year females (bias = 0.00).

In contrast, the performance of the two linear models were weak (Fig. 5). When all data without accounting for sex and age were used, the estimates of the linear model suggested that the average wing length of captures increased over the years. This is because due to the assumed shift in phenology of captured individuals, the proportion of captured adults increased over the years. In contrast, when only the identified first year females were used in the linear model, the estimate was not biased but the credible intervals were much large compared to the credible intervals obtained using our approach. This low precision of estimates in the linear model is because the sex and age of only a fraction of the first year females was identified and, thus, only few data could be used in the linear model.
